# The Importance of Biotic vs. Abiotic Drivers of Local Plant Community Composition Along Regional Bioclimatic Gradients

**DOI:** 10.1371/journal.pone.0130205

**Published:** 2015-06-19

**Authors:** Kari Klanderud, Vigdis Vandvik, Deborah Goldberg

**Affiliations:** 1 Department of Ecology and Natural Resource Management, Norwegian University of Life Sciences, Ås, Norway; 2 Biology Department, University of Bergen, Bergen, Norway; 3 Department of Ecology and Evolutionary Biology, University of Michigan, Ann Arbor, Michigan, United States of America; Institute of Ecology and Biodiversity, CHILE

## Abstract

We assessed if the relative importance of biotic and abiotic factors for plant community composition differs along environmental gradients and between functional groups, and asked which implications this may have in a warmer and wetter future. The study location is a unique grid of sites spanning regional-scale temperature and precipitation gradients in boreal and alpine grasslands in southern Norway. Within each site we sampled vegetation and associated biotic and abiotic factors, and combined broad- and fine-scale ordination analyses to assess the relative explanatory power of these factors for species composition. Although the community responses to biotic and abiotic factors did not consistently change as predicted along the bioclimatic gradients, abiotic variables tended to explain a larger proportion of the variation in species composition towards colder sites, whereas biotic variables explained more towards warmer sites, supporting the stress gradient hypothesis. Significant interactions with precipitation suggest that biotic variables explained more towards wetter climates in the sub alpine and boreal sites, but more towards drier climates in the colder alpine. Thus, we predict that biotic interactions may become more important in alpine and boreal grasslands in a warmer future, although more winter precipitation may counteract this trend in oceanic alpine climates. Our results show that both local and regional scales analyses are needed to disentangle the local vegetation-environment relationships and their regional-scale drivers, and biotic interactions and precipitation must be included when predicting future species assemblages.

## Introduction

Consequences of climate change for species assemblages and biodiversity may depend on how the relative importance of biotic and abiotic interactions shifts with the environment. Thus, disentangling the relative impact of biotic and abiotic factors on species composition, and how these vary with environmental conditions, are urgently needed to understand how climate change affects ecological processes and biodiversity across regions [1]. This is a recent restatement of the classical debate within ecological theory about the determinants of plant community species composition [2, 3]: Abiotic factors, such as soil moisture, nutrients and pH are traditionally predicted to more directly affect species establishment and survival under stressful environmental conditions, such as in the alpine [4]. Biotic interactions, on the other hand, such as competition from denser and higher vegetation and a larger amount of litter, are often predicted to play a more important role for species coexistence in more productive habitats [5, 3, 6, 7, 8], but see [9, 10, 11]. Negative biotic interactions are also found in stressful environments (e.g. [12]), whereas more recently, a number of studies have documented significant impacts of positive biotic interactions for plants in cold, dry, or infertile unproductive habitats [13, 14, 15, 16, 17, 18]. Thus, biotic interactions may be equally important in stressful and more productive habitats, although the relative importance of different types of biotic interactions (positive vs. negative) may shift [19].

Empirical assessment of trends in the impact of species interactions, whether negative or positive, along broad-scale productivity or stress gradients are typically based on meta-analyses of experimental manipulation of neighbours and quantification of responses in terms of plant growth, or less commonly, survival [20, 16]. However, such individual-level data cannot necessarily be scaled up to consequences of these interactions at community-level. To take an extreme (and unlikely) example: if all species were strongly reduced in growth and survival by competition, but suffered similar reductions, their relative abundance would not be affected [21]. Thus, approaches to assess effects of biotic interactions on entire communities are needed.

In this paper we take a non-experimental approach disentangling the relative importance of interactions among plants vs. the abiotic environment for structure of entire communities. We first assess the variation in fine-scale vegetation accounted for by local biotic vs. abiotic variables, where the biotic variables are proxies for the intensity of interactions, such as the cover or the height of vegetation. We then compare the relative variation accounted for by local biotic vs. abiotic variables across sites that differs substantially in environmental stress and productivity. While this observational approach cannot unambiguously isolate cause and effect, it does avoid experimental artefacts and can more easily focus on the entire community rather than simple measures of individual growth for one or a few component species. The use of observational, rather than experimental data also facilitates comparison across different sites and scales.

Hierarchical multivariate variation partitioning approaches [22], see also [23] are often used to assess the relative importance of different local- and regional-scale environmental factors for community composition (e.g. [24, 25, 26, 27]). However, these approaches estimate variation without taking into account differences in responses across environmental gradients, i.e. all local communities may not respond to the same underlying environmental factors, and they may not respond in the same way to those factors. We hypothesize that the relative importance of biotic and abiotic factors, and possibly the direction of biotic effects (positive vs. negative), differs between local communities. Thus, we supplement the variation partitioning approach with replicated local-scale ordination analyses to allow assessment of the relative importance of different environmental factors within each local community along broader-scale climate gradients. In our study, we thus combine broad- and fine-scale ordination analyses of vegetation data from local sites within a regional-scale grid of sites to compare the effect of biotic vs. abiotic factors along environmental gradients.

The impacts of biotic and abiotic factors on plant communities along elevation gradients have primarily been studied, or interpreted, as temperature gradients [16], but see [28]. Temperature and precipitation are, however, often correlated along elevation gradients, and recent studies have pointed at the importance of precipitation, soil moisture, and their interactions with temperature and local environmental factors as drivers of community dynamics [29, 30, 31]. Both temperature and precipitation regimes have changed worldwide during the last century, and are predicted to increase by 2.3–4.6°C and 5–30%, respectively, in Northern Europe towards 2100 [32]. Towards higher elevations, there is also an increasing harshness and decreasing productivity. Higher temperatures and precipitation rates may increase productivity in these areas and thereby alter the relative role of biotic and abiotic interactions. In accordance with this, vegetation canopy height and litter cover have increased in alpine and arctic plant communities during the last 20–30 years, both in climate warming experiments [33, 29] and in unmanipulated monitoring plots [30]. These studies and others (e.g. [34]), also show that graminoids have increased more in absolute abundance than forbs, especially in sites with high ambient site temperature and soil moisture [30], and forbs decrease in absolute abundance in the warmest sites [30]. Graminoids are better competitors for nutrients and light than most forbs [35], and consequently, the decline of forbs may be due to biotic interactions rather than a direct climate effect. This suggests that climate warming and/or increased precipitation may directly benefit graminoids, whereas biotic interactions caused by graminoid dominance, may decrease forb abundance.

To separate the effects of temperature and moisture on the relative importance of biotic and abiotic factors, we established twelve study sites along natural temperature and precipitation gradients in southern Norway, such that these two main climate variables varied independently (Table 1) and all factors other than climate were as identical as possible; including vegetation type, bedrock, aspect, slope, and land use [36]. Within each site in this regional ‘climate grid’, we sampled vegetation and associated biotic and abiotic environmental conditions on a fine-scale.

**Table 1:** Altitude, annual precipitation, summer temperature, number of plots sampled and mean ± standard deviations of predictor variables at alpine, sub alpine, and boreal grassland sites along precipitation gradients (low [1] to high [4]) in southern Norway.

Site	Site name	Altitude (m a.s.l.)	Precip (mm)	Temp (°C)	No plots	pH	LOI (%)	Moisture (%)	Vegetation height (cm)	Litter cover (%)	Bryophyte cover (%)
ALPINE 1	Ulvhaugen	1208	596	6.17	20	5.7±0.2	7.9±2.4	37.5±5.7	3.6±1.1	17.6±7.1	13.0±13.7
ALPINE 2	Låvisdalen	1097	1321	6.45	20	5.9±0.2	8.2±2.7	40.0±5.4	3.5±1.3	12.4±3.4	17.1±8.5
ALPINE 3	Gudmesdalen	1213	1925	5.87	19	6.1±0.2	15.3±4.3	50.8±6.9	4.6±2.2	15.0±5.5	20.4±25.4
ALPINE 4	Skjellingahaugen	1088	2725	6.58	10	6.1±0.6	7.1±3.8	47.6±6.6	4.1±0.9	12.4±6.9	28.0±16.9
SUB-ALPINE 1	Ålrust	815	789	9.14	20	5.4±0.2	9.1±3.1	33.1±5.2	3.3±1.2	11.3±4.1	17.1±17.1
SUB-ALPINE 2	Høgsete	700	1356	9.17	20	5.2±0.2	12.9±0.8	36.5±1.5	8.9±2.8	13.2±6.2	34.9±14.5
SUB-ALPINE 3	Rambæra	769	1848	8.77	18	5.4±0.3	10.0±3.2	37.5±7.4	5.3±2.5	11.4±4.0	29.9±17.8
SUB-ALPINE 4	Veskre	797	3029	8.67	10	5.8±0.2	13.4±2.5	50.2±3.8	4.1±1.2	8.2±3.2	25.4±17.2
BOREAL1	Fauske	589	600	10.3	10	5.3±0.1	10.7±1.4	34.8±3.0	9.4±2.6	15.3±5.5	9.7±6.9
BOREAL 2	Vikesland	474	1161	10.55	10	5.2±0.2	14.7±2.1	35.0±2.8	8.6±4.1	16.1±7.5	50.1±20.1
BOREAL 3	Arhelleren	431	2044	10.60	10	5.2±0.1	10.1±1.3	43.8±2.0	15.9±5.2	10.2±2.8	41.9±22.9
BOREAL 4	Øvstedal	346	2923	10.78	5	5.3±0.2	10.1±3.7	31.5±5.7	11.9±2.8	9.1±4.1	40.7±22.8

LOI refers to Loss-On-Ignition, a measure of soil organic matter.

We used these data to test the following hypotheses: 1) The community response to local-scale variation in biotic and abiotic variables is not consistent across broad-scale bioclimatic gradients. 2) The local abiotic environment accounts for more variation in species composition in stressful and unproductive alpine and dry sites than in the more benign and productive boreal and mesic sites. 3) Biotic variables account for more variation in species composition in warmer and wetter sites. 4) Forbs are more strongly affected by biotic interactions than graminoids. We conclude by discussing the implications of these results for grassland species composition under warmer and wetter future climates. Our results show that the importance of abiotic vs biotic variables for species composition shift along the broad scale temperature and precipitation gradients and we propose that both local and regional scales analyses is crucial to be able to detect and disentangle the different drivers of local vegetation-environment relationships.

## Materials and Methods

**Fig 1:**
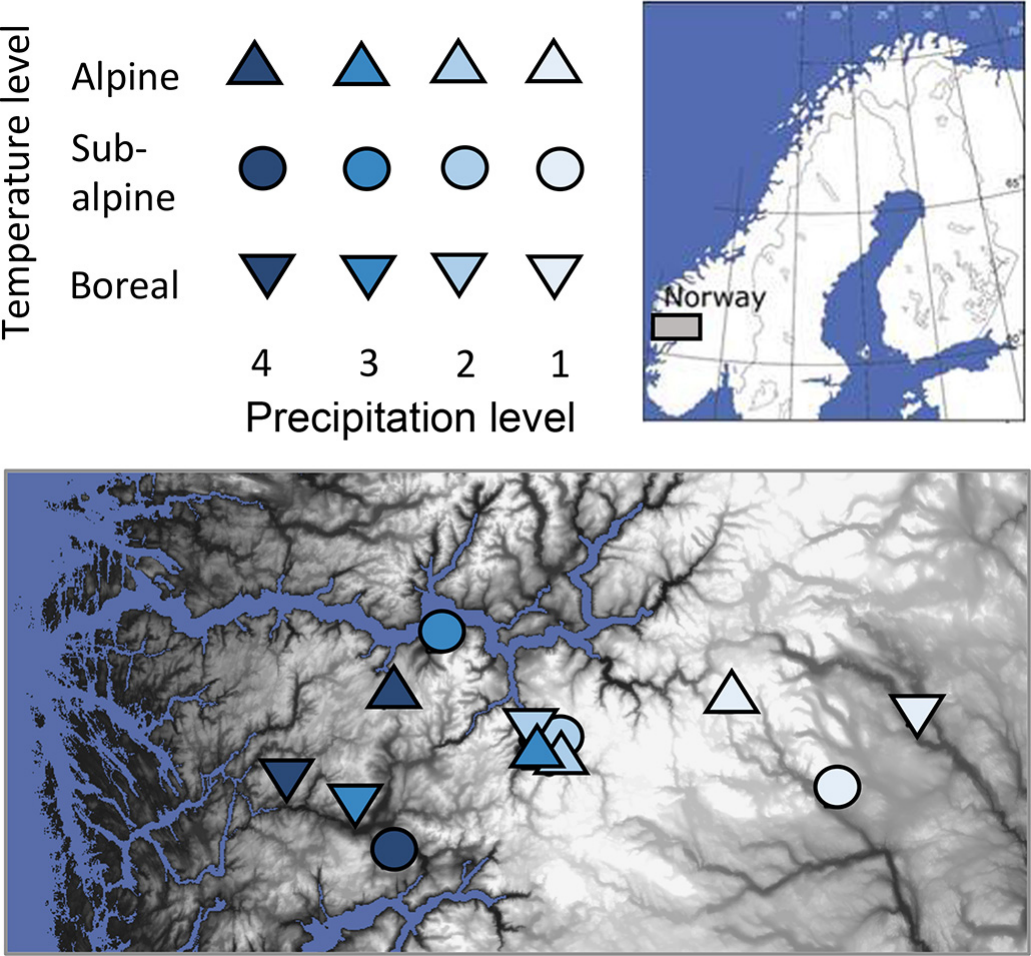
Map and study design. Location of the twelve study sites along temperature and precipitation gradients in the fjord landscape of southern Norway.

The study was conducted at twelve sites situated from the continental east to the oceanic west and from the alpine to the boreal climatic zones in the fjord landscape of southern Norway. The sites are distributed across a unique climate grid with three levels of summer temperature (mean of four warmest months) replicated at four levels of mean annual precipitation (Fig 1, Table 1). The climate data are interpolated with 100 m resolution [37] from the normal period 1961–1990 [38]. The sites were selected to be as similar as possible with respect to all factors other than climate to facilitate comparisons between sites. The sites are all grasslands associated with calcareous bedrock. Most of the sites are south-west exposed slopes of ca twenty degrees inclination, except one (Boreal 3) that is east exposed. All sites are moderately grazed. Geographical distance between sites is on average 15 km and ranges from 175 km (Boreal 1 and Boreal 4) to 650 m (Boreal 2 and Sub alpine 2; these are also 400 m a.s.l. apart). Within sites, all plots were situated in the selected grassland within 5 blocks with a total area of ca. 75–200 m^2^. All the plant communities are within the plant sociological association Potentillo-Festucetum ovinae [39]. In the alpine, this type tends towards Potentillo-Poligonium vivipari, and in some of the lowland sites, they tend towards Nardo-Agrostion tenuis [39].

Vegetation sampling was conducted in 25 x 25 cm plots randomly positioned within five blocks at each site. The number of plots differs between sites due to the design of a transplant experiment conducted between the sites (Table 1). We estimated percentage cover of all vascular species, and the total cover of bryophytes, litter, and bare soil in each plot. We used a ruler at four fixed points in each plot to measure the mean height of the vegetation. All of these variables are important proxies for the intensity of positive as well as negative biotic interactions among plants in boreal and alpine grasslands. Height of the vascular vegetation may indicate the intensity of competition for light. Increasing vascular cover may also indicate increasing competition (for light or belowground), but under harsh environmental conditions increasing cover has also been shown to facilitate recruitment [40, 41] and plant growth [35]. Bryophytes may limit seedling emergence and growth by limiting access to soil, shading or allelopathic interactions [15, 42], limit growth of the vascular vegetation [43], or facilitate other species due to their water holding capacities [44]. Similarly, litter cover may limit plant recruitment by shading, act as a physical barrier to seedlings and shoots (e.g. [45, 46], or facilitate plant growth by providing nutrients through decomposition or by ameliorating drought or temperature extremes [47]. All the sites are on non-protected privately owned land, and permits for doing field sampling has been given from the twelve landowners. No protected species were sampled. The sampled vegetation data are deposited in the Nordic vegetation database (NVD, http://www.givd.info/ID/EU-00-018) [48].

To quantify the local abiotic environment, we measured soil pH, moisture, and organic content (proxy for nutrient availability) in each plot. These are important determining factors for plant species composition and diversity in this system [24, 40]. We were able to take the soil samples from directly underneath the sampled vegetation in each of the plots, because, after the vegetation was sampled, all plots were dug up and transplanted to a new site for another experiment. The soil samples were stored cold and brought to the freezer directly after sampling. Before analyses the soil was thawed and put through a two mm sieve. Soil pH was measured after adding 50 ml distilled water to 25 g soil and mixing for two hours. To measure water content, the soil was weighed, and then dried at 105°C for 24 hours and left one hour in a desiccator before weighing again. Loss-on-ignition (LOI) was used to estimate soil organic matter; the soil was burnt for another six hours at 550°C and then left one hour in the desiccator before weighing. We also measured soil moisture in the field by using a SM 200 Soil moisture sensor (Delta-T Devices Ltd, UK) at four fixed points per plot and calculated the average. Soil moisture was positively correlated between the soil sampled from the field and the direct field measurements, and to obtain more sampling points on a time scale, we used the average of the soil sample and field measurements in the analyses.

### Statistical analyses

To determine whether the design of the grid with orthogonal temperature and precipitation gradients corresponded to clear vegetation patterns with respect to these two factors, we first examined the regional-scale pattern in vegetation-environment relationships by means of a canonical correspondence analysis (CCA; [49]) with all sites, climate variables, and local environmental variables included. Unimodal-based methods were chosen because the gradients in the overall compositional data were relatively strong (gradient length axis 1 = 3.69, axis 2 = 2.5, axis 3 = 3.32, axis 4 = 2.30 SD, assessed by detrended correspondence analysis with default options).

We then partitioned the community data into graminoid and forb species composition and conducted a series of analyses on these two datasets to determine the relative importance of the biotic and abiotic variables for local community composition within each dataset along the bioclimatic gradients.

First, a standard hierarchical variation partitioning [22], see also [23] was used to assess the overall importance of the different groups of explanatory variables on regional (among-site) and local (within-site) scales. This was done in three steps: (1) We assessed the total variation accounted for by each local abiotic (soil moisture, pH, LOI) or biotic (vegetation height and cover, bryophyte cover, litter cover, bare soil) variable. For the variable ‘vegetation cover’, the sum of all forb species’ covers was used as a predictor for the graminoid dataset, and the sum of all graminoid species’ covers was used as a predictor for the forb dataset. Similarly, we quantified the variation explained by each of the the two groups (abiotic, biotic) where only significant (p < 0.05) variables, as assessed by forward selection, were retained. (2) For each of the analyses above, variation at the within-site or local scale was quantified by running the analyses described above with dummy variables representing all the twelve sites as co-variables in partial CCAs. (3) Variation at the among-site or regional scale was calculated by subtracting this local-scale variation (calculated in step 2) from the total variation explained by that variable or group (calculated in step 1).

Second, to test whether variation explained by biotic and abiotic variables differed among the 12 local sites (i.e., hypothesis 1) we conducted a series of site-wise ordination analyses. As it could be easier to pick up biotic or abiotic driven variability in more heterogeneous datasets, we used multivariate dispersion to statistically compare the magnitude of within-site heterogeneity in species composition among sites for multivariate species compositional data [50]. This analysis shows that the within-site compositional variability is comparable in magnitude among sites (multivariate dispersion: 0.37±0.04), and any differences were unrelated to the broad-scale temperature and precipitation gradients (temperature; r^2^ = 0.05; precipitation: r^2^ = 0.02). The species composition within each site was relatively homogenous (gradients < 2.5 SD, assessed by detrended correspondence analyses), and we therefore used redundancy analysis (RDA; [51]) to assess the proportion of variation in graminoid and forb species composition explained by abiotic and biotic factors for each of the sites. Site Boreal 4 was removed from the data before analyses due to low sample size. For each of the 11 remaining sites, we then estimated the variation in the graminoid and forb species composition explained by each explanatory variable both separately (8 variables, graminoids and forb community, 11 sites; total of 176 ordination analyses) and jointly for the two groups of explanatory variables (biotic vs abiotic; two communities, 11 sites for a total of 44 ordination analyses). In the group-wise ordination analyses we used forward selection within each dataset, and included only significant variables (p < 0.1) in each of the two groups, or, if none were significant, the best predictor in each group, in the final analyses. We used presence/absence data and downscaling of rare species in all ordinations, which, along with the forward selections, were conducted in Canoco 5 [52].

We analysed the output from these site-wise ordination analyses to test whether the relative importance of biotic vs. abiotic variables in determining species composition changes in consistent ways along temperature and precipitation gradients (hypothesis 2, 3), or varies between the functional groups (graminoids, forbs; hypothesis 4). We first fitted simple linear regressions to assess if the variation in forb or graminoid species composition explained by each of the local environmental variables (individual biotic and abiotic variables, quantified in the site-wise analyses described above) varied systematically along the regional-scale temperature and precipitation gradients. We then tested the relative explanatory power of biotic and abiotic factors for local community patterns more formally in an analysis where the proportion of the total explained variation accounted for by the biotic variables in the RDA analyses were used as response variable in an ANOVA with temperature (alpine, sub alpine, boreal), precipitation (levels1-4), or functional group (forb or graminoid), and their interactions as explanatory variables. Proportional data were used in these overall models to avoid biases due to methodological artefacts causing different fractions of the total variation explained among datasets [53]. These analyses were performed in R version 2.15.3 [54] using RStudio version 0.96.331 (RStudio, Inc., Boston, Massachusetts, USA) and the car package [55].

## Results

**Fig 2:**
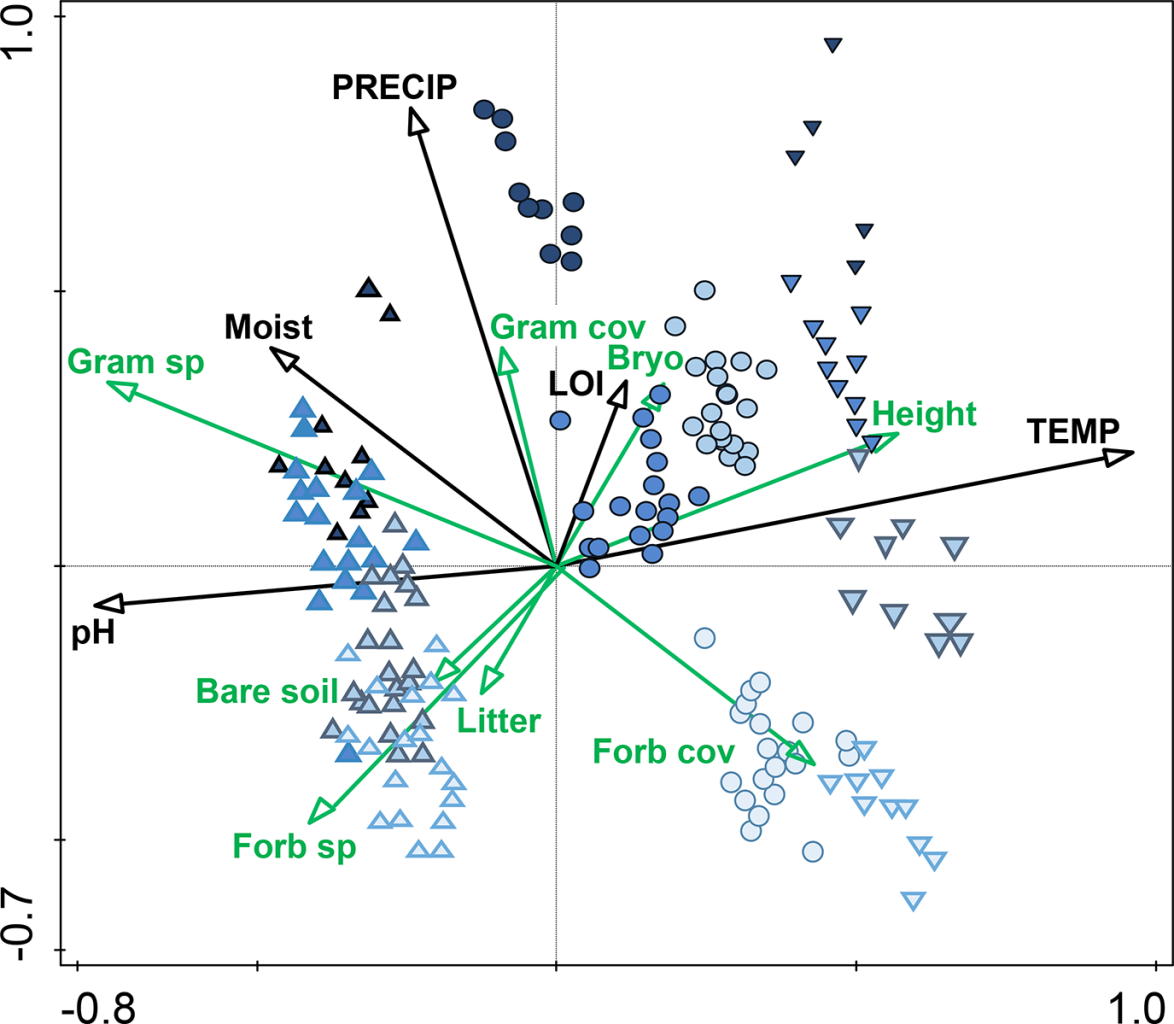
Overall ordination of the grid. Canonical correspondence analysis (CCA) of measured biotic (green) and abiotic (black) environmental variables and alpine (▲), sub-alpine (●) and boreal (▼) sites along a precipitation gradient (1–4 from light to dark blue, see Fig 1) in southern Norway. LOI refers to Loss-On-Ignition, a measure of soil organic matter. Eigenvalues axis 1 = 0.472, axis 2 = 0.248, axis 3 = 0.182, axis 4 = 0.137. Only the two first axes are shown.

The canonical correspondence analysis (CCA) shows that the main patterns in plant community composition in the full dataset reflect the temperature (axis 1) and precipitation (axis 2) of the sites distributed within the climate grid in southern Norway (Fig 2). The site mean biotic and abiotic environmental variables are also related to these major gradients. As expected, vegetation height increases towards warmer climates, soil moisture increases with precipitation, whereas pH is higher in alpine sites. Interestingly, graminoid cover and richness increase with precipitation and soil moisture, whereas forb cover is negatively correlated with these variables and with the graminoids (Fig 2). Bare soil, indicating disturbance or bad conditions for plant growth, is most common in dry alpine sites, and highly correlated with forb species richness. Bryophyte cover on the other hand, is negatively correlated with forb species richness, and, together with soil organic content (LOI), highest towards warmer and wetter sites.

**Fig 3:**
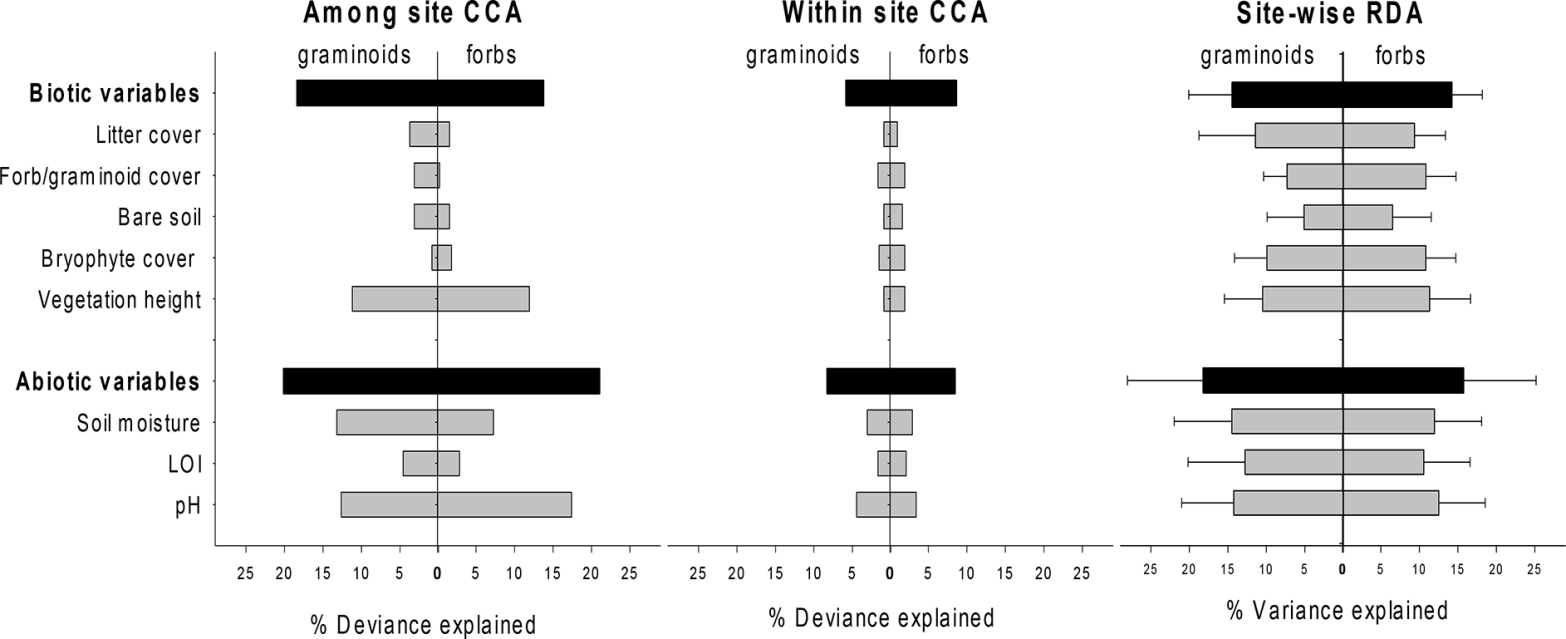
Variation explained at broad and fine geographical scales. Compositional variation in graminoid and forb species (% of total deviance [CCA] or variation [RDA]) explained by abiotic and biotic environmental variables, grouped and separately, at the a) regional/among site scale and b) local/within site scale when all sites are part of the same canonical correspondence analysis (CCA). Part c) shows parallel results for separate site-wise redundancy analyses (RDA) analyses for each of eleven grassland sites in southern Norway. LOI refers to Loss-On-Ignition, a measure of soil organic matter.

These regional-scale patterns are reflected in the among-site variation components in both the graminoid and the forb species composition (Fig 3A). Vegetation height, soil moisture and pH are the most important variables for both functional groups, with soil moisture somewhat more important for graminoids, and pH more important for forbs. The low explanatory power of all environmental variables at the local scale (Fig 3B; abiotic variables average 3.17% ± 1.38 for graminoids, 2.82 ± 0.66 for forbs; biotic average 1.20% ± 0.38 for graminoids, 1.69 ± 0.42 for forbs) suggests that either the measured variables are not important for species composition within sites, or that the community responses to these variables differ between sites.

**Fig 4:**
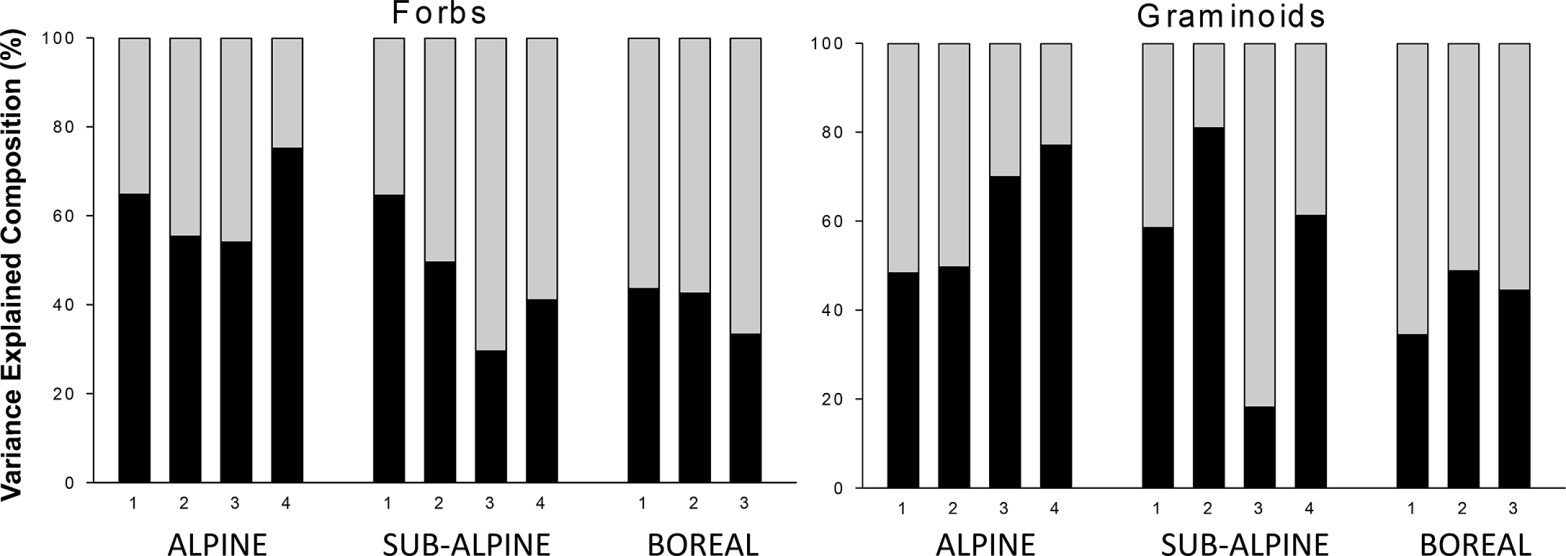
Variation explained by abiotic vs. biotic variables. Relative variation in species composition explained by abiotic (black) and biotic (grey) environmental variables in site-wise redundancy analyses (RDA) performed across eleven grassland sites in alpine, sub alpine and boreal sites along a precipitation gradient from low (1) to high (4) in southern Norway. LOI refers to Loss-On-Ignition, a measure of soil organic matter.

To distinguish between these possibilities, vegetation-environment relationships were analysed separately for each site. The explanatory power of both biotic and abiotic variables for local compositional patterns increases substantially in the site-wise analyses relative to the analyses on the entire dataset (Fig 3C; abiotic variables average 13.83% ± 0.93 for graminoids, 11.69 ± 1.00 for forbs; biotic average 8.85% ± 2.58 for graminoids, 9.78 ± 1.96 for forbs, across sites). The regression slopes of the linear models indicate that this local explanatory power varies systematically between biotic and abiotic variables, along the climate gradients, and between forbs and graminoids (Table 2). The explanatory power of individual local biotic variables changes more consistently in response to temperature than the abiotic variables: All biotic variables have consistently positive (if rarely statistically significant) regression slopes and thus likely explain more of the variation in both forb and graminoid species composition towards warmer sites (Table 2). As expected, forbs have steeper regression slopes for the biotic variables than graminoids. The patterns along the precipitation gradient are less clear, but soil pH explains more of graminoid species composition towards wetter sites (Table 2). The ANOVA results confirm these patterns–the proportion of the total explained variation in the site-wise RDAs explained by the biotic variables (relative to the abiotic) increases systematically with increasing temperature, and a significant interaction with precipitation suggests that the relative role of local biotic and abiotic environmental variables shifts along the broad scale climate gradients (Table 3, Fig 4). Moreover, biotic variables seem to explain more towards wetter sub alpine and boreal sites, but more towards dryer sites in the alpine (Table 3, Fig 4).

**Table 2:** Regression slopes and associated p-values (* *p* < 0.05, · *p* < 0.1, no symbol = not significant) of relationships between variation in forb and graminoid species composition explained by local abiotic and biotic environmental variables in redundancy analyses (RDA) of local grassland species composition along broad scale temperature and precipitation gradients in southern Norway.

Temperature			Precipitation	
	Forbs	Graminoids	Forbs	Graminoids
Abiotic variables	- 3.153*	- 1.852	+ 0.339	+ 0.596
pH	- 0.067	- 0.859	+ 0.323	+ 0.521*
LOI	+ 0.072	+ 0.857	+ 0.078	+ 0.095
Moisture	+ 0.411	- 0.898	- 0.136	+ 0.347
Biotic variables	+ 0.254	+ 1.191	+ 0.215	- 0.056
Vegetation height	+ 1.318	+ 1.227	+ 0.196	+ 0.109
Litter cover	+ 1.222·	+ 0.978	+ 0.164	- 0.107
Bryophyte cover	+ 1.040·	+ 1.025	+ 0.099	+ 0.029

LOI refers to Loss-On-Ignition, a measure of soil organic matter. n = 11.

**Table 3:** ANOVA estimates, F- and P-values of the relative proportion of the explained variation accounted for by biotic (vs abiotic) variables in redundancy analyses (RDA) of graminoid and forb species composition in grassland sites along temperature and precipitation gradients in southern Norway.

Predictors	df	Estimates	F	P
Intercept		43.45		
Temperature	2		6.91	0.011
Sub-alpine		-4.99		
Boreal		17.54		
Precipitation	3		2.71	0.096
2		4.05		
3		-5.44		
4		-19.55		
Temperature × Precipitation	5		3.57	0.037
Sub-alpine × 2		-7.75		
Boreal × 2		-10.73		
Sub-alpine × 3		43.12		
Boreal × 3		5.56		
Sub-alpine × 4		29.94		

Temperature and precipitation are expressed factorial variables (three temperature levels: alpine, sub-alpine, boreal, and four precipitation levels 1–4). Functional type (graminoid vs forb) and its interactions were not significant and therefore not included in the final model. n = 22.

## Discussion

Vegetation and environmental analyses of alpine and boreal grasslands of southern Norway show that the abiotic and biotic drivers of local-scale variation in plant community composition shift along the regional-scale temperature and precipitation gradients. Although the patterns are not strong, our results to some extent support the predictions of a decreasing role of abiotic and an increasing role of biotic environmental variables for species composition towards warmer climates (e.g. [4, 5, 3]). Interactions between temperature and precipitation indicate, however, that the importance (as measured by variation explained) of biotic vs. abiotic variables varies in complex ways, in some cases driven by the specific biotic or abiotic variable.

The shift between local abiotic and biotic determinants of species composition towards warmer sites reflects the productivity gradient in the region, with plant community biomass increasing from alpine to boreal sites [56]. Thus, our results are in line with the predictions that in the alpine, where vegetation cover is sparser, the impacts of the abiotic environment is most important for species composition, whereas in the lowlands where plant biomass is higher, effects of the biotic environment are more prevalent. Nevertheless, although the role of local biotic environmental variables slightly increased towards warmer and more productive sites in our study area, precipitation gradients showed no consistent trend and even the temperature pattern was relatively weak and noisy. We suggest that this is because biotic interactions represent the net effects of both facilitative and competitive processes, with the role of competition predicted to decrease, and facilitation to increase, towards stressful environments (the stress gradient hypothesis; [57, 13, 16]). Although our study cannot distinguish between competitive and facilitative processes, our results suggest that biotic interactions are important for plant community structure also in cold, low-productivity systems. Competitive interactions have been shown to affect recruitment also in alpine [58, 59, 41] and arctic [15] plant communities. In line with these findings, other studies in our climate grid have also shown that the vegetation canopy limits seedling recruitment both in alpine and boreal sites, although less pronounced in the alpine [60]. On the other hand, an intact vegetation canopy, and a dense litter and bryophyte cover, may also facilitate plant recruitment and growth in the climatically severe alpine sites by providing, shelter, nutrients or moisture [13, 16, 18].

The biotic variables tended to explain a higher proportion of the variation in forb than of graminoid species composition towards warmer sites in the study area. Forbs may be more susceptible to biotic interactions than graminoids, and the negative correlation in the overall ordination in Fig 1 between forb cover and graminoids also indicates a competitive relationship between the two functional groups, which is in line with results from a graminoid removal experiment performed in the same grid of sites. This may suggest that the presence of graminoids in the community can decrease forb cover, in particular when precipitation and soil moisture increase [56]. Increased abundance of graminoids at the expense of forbs is found in experiments where warming and nutrient addition generally benefit graminoids more than forbs [61, 62, 63, 36]. This is likely because graminoids respond faster than most of the forbs to the nutrients added [62], and thereby out-compete the low-stature forbs for space and light by their higher standing biomass. In addition to the height of the vegetation, litter and bryophyte cover also seemed to be more important determinants of forb than of graminoid composition towards warmer sites. Both litter [46] and bryophytes [15, 58, 42] may limit seedling recruitment, and could therefore act more as limiting factors for forbs than for graminoids, as forbs generally depend more on seedling recruitment for population persistence than the more clonal graminoids. Moreover, a thick moss cover, as found in the warmest sites in our study, may have negative effects on low-stature forbs through overgrowth of stems and leaves [64, 65, 43] or through allelopathic interactions [42]. Decay processes may also be faster and more prevalent in warm sites, and nutrients made available from decomposing litter may benefit graminoids more than forbs. Thus, these local biotic variables may have different effects on forbs and graminoids, respectively, due to differences in recruitment and growth traits between these functional groups.

The patterns along the precipitation gradient were generally complex, with precipitation effects on the relative role of local biotic and abiotic variables depending on temperature (i.e., differing between alpine, sub alpine and boreal sites). The relationship between plant community biomass and precipitation is unimodal throughout the study area, with highest productivity at intermediate precipitation levels [56]. The role of the local biotic environmental variables for species composition slightly followed this pattern, whereas abiotic variables explained, as expected, a lesser proportion of the variation in species composition towards wetter and more productive sub alpine and boreal sites. In the alpine on the other hand, abiotic variables explained more towards wetter sites even though productivity is higher here than in the dryer alpine sites. This might be explained by the large amounts of the precipitation falling as snow in the wet alpine sites, resulting in shorter growing seasons and consequently a more stressful environment for the plants. Hence, snow cover likely overrules the effects of biotic interactions for plant species composition in snow rich areas.

Our results from analyses of local-scale vegetation patterns across a climate grid in southern Norway strongly support recent studies that call for vegetation-environment analyses performed across different spatial scales [18, 66, 67]. As the importance of different variables for species composition shift along the bioclimatic gradients in our study system, analyses on both local and regional scales are needed to detect and disentangle the local vegetation-environment relationships and their regional-scale drivers. Surrogates of biotic interactions, in particular the height of the vegetation, explained a substantial proportion of both alpine, sub alpine, and boreal species composition, with an increasing role towards warmer sites. This suggests that biotic interactions may become more important for alpine and boreal grassland community structure and composition in the future. In oceanic alpine regions, this trend may be counteracted by increased winter precipitation, resulting in stronger abiotic environmental control. Based on our results we also hypothesise that graminoids might benefit more from warmer and wetter future climates than forbs, and that forbs might decline due to competition from increasing abundances of graminoids in the future [61, 62, 35, 34]. Our results highlight the importance of including biotic interactions in models predicting future distribution of species assemblages [66], and that precipitation needs to be taken into account in climate change effects studies.
